# Protein-Centric Analysis of Personalized Antibody Repertoires Using LC-MS-Based Fab-Profiling on a timsTOF

**DOI:** 10.1021/jasms.4c00076

**Published:** 2024-04-25

**Authors:** Jan Fiala, Dina Schuster, Simon Ollivier, Stuart Pengelley, Markus Lubeck, Florian Busch, Andris Jankevics, Oliver Raether, Jean-Francois Greisch, Albert J. R. Heck

**Affiliations:** † Biomolecular Mass Spectrometry & Proteomics, Bijvoet Center for Biomolecular Research & Utrecht Institute for Pharmaceutical Sciences, Utrecht University, Padualaan 8, 3584 CH Utrecht, The Netherlands; ‡ Netherlands Proteomics Center, Padualaan 8, 3584 CH Utrecht, The Netherlands; § Bruker Daltonics GmbH & Co. KG, Fahrenheitstrasse 4, 28359 Bremen, Germany; ∥ Bruker Switzerland AG, 8117 Fällanden, Zurich Switzerland

**Keywords:** antibody repertoires, serum proteomics, protein-centric proteomics, personalized humoral immune response, timsTOF

## Abstract

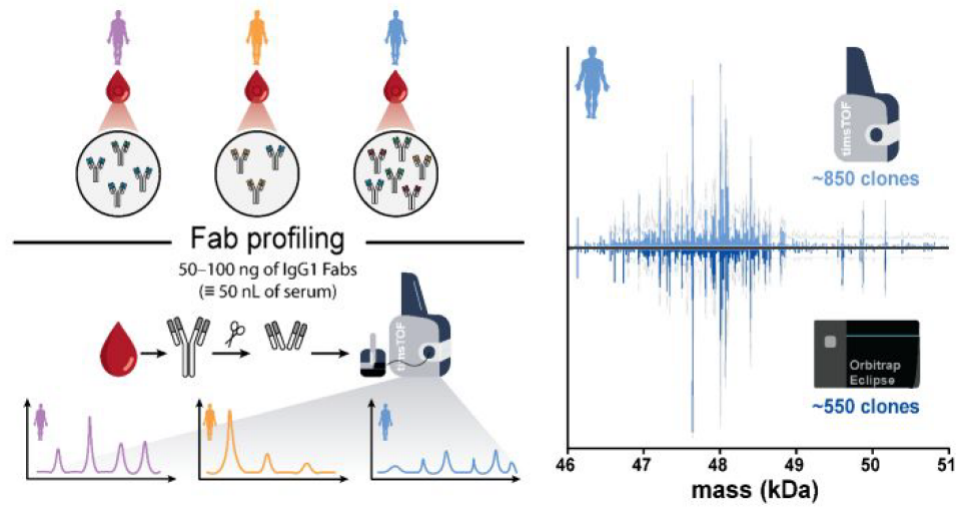

Endogenous antibodies, or immunoglobulins (Igs), abundantly present in body fluids, represent some of the most challenging samples to analyze, largely due to the immense variability in their sequences and concentrations. It has been estimated that our body can produce billions of different Ig proteins with different isotypes, making their individual analysis seemingly impossible. However, recent advances in protein-centric proteomics using LC-MS coupled to Orbitrap mass analyzers to profile intact Fab fragments formed by selective cleavage at the IgG-hinge revealed that IgG repertoires may be less diverse, albeit unique for each donor. Serum repertoires seem to be dominated by a few hundred clones that cumulatively make up 50–95% of the total IgG content. Enabling such analyses required careful optimization of the chromatography and mass analysis, as all Fab analytes are highly alike in mass (46–51 kDa) and sequence. To extend the opportunities of this mass-spectrometry-based profiling of antibody repertoires, we here report the optimization and evaluation of an alternative MS platform, namely, the timsTOF, for antibody repertoire profiling. The timsTOF mass analyzer has gained traction in recent years for peptide-centric proteomics and found wide applicability in plasma proteomics, affinity proteomics, and HLA peptidomics, to name a few. However, for protein-centric analysis, this platform has been less explored. Here, we demonstrate that the timsTOF platform can be adapted to perform protein-centric LC-MS-based profiling of antibody repertoires. In a side-by-side comparison of the timsTOF and the Orbitrap we demonstrate that the extracted serum antibody repertoires are alike qualitatively and quantitatively, whereby in particular the sensitivity of the timsTOF platform excels. Future incorporation of advanced top-down capabilities on the timsTOF may make this platform a very valuable alternative for protein-centric proteomics and top-down proteomics and thus also for personalized antibody repertoire profiling.

## Introduction

Antibodies or immunoglobulins (Igs) are large biomolecules used by the immune system to help recognize and clear harmful materials, such as viruses and bacteria. Their high specificity for their targets (antigens) also makes them popular biotherapeutics. Igs are abundantly present in different body fluids, such as serum, saliva, milk, and mucosal linings, as well as cerebrospinal fluid, blister fluids, and even tears.^1^ Detecting and characterizing—or profiling—the antibody repertoire can help to better understand immune responses and immune defects and guide therapeutic development.

A variety of technologies exist to profile humoral immune responses; however, many of these target integral antibody responses and not the antibody clonal population. Commonly used tools include solid surface assays with immobilized antigens (ELISA) or phage display systems, such as phage-display immunoprecipitation sequencing (PhIP-Seq).^2^ These assays can be performed in a high-throughput manner, enabling the screening of several thousands of antigens to extract information on the antibody reactivity. While these techniques are helpful to study individual immune responses and reactivity to antigens, they fail to recapitulate the diversity of the underlying antibody population and do not provide insights into individual clones. Next-generation sequencing approaches can give insights into the clonality of a B-cell receptor at the gDNA or mRNA level,^3^ but they do not directly provide qualitative and quantitative information on the genuine circulating antibody population.

To address this caveat, mass-spectrometry-based approaches have been introduced that offer solutions to some of these issues.^4^ Bottom-up proteomics can be used for quantitative analysis of complex samples; however, the sequence coverage and therefore also the profiling depth that is achievable with bottom-up approaches are still limited. In the case of real-life biological samples, such as serum or milk (and mixtures of antibodies), the specific origin of the identified peptides may easily be lost, as most of the Ig clones are very similar in sequence. Top-down proteomics analyzes intact antibodies but is mostly limited to the identification of highly abundant (and or highly purified) Igs and suffers from limited throughput compared to genomic sequencing and bottom-up proteomics.^5^ An alternative is to use middle-down proteomics, a method whereby Igs are enzymatically cleaved at the hinge region to separate the fragments antigen-binding (Fab) from the fragment crystallizable (Fc) region. Studying Fabs instead of intact Igs is an interesting option as the Fab domains are chiefly responsible for the antigen specificity of the Igs. This middle-down approach yields improved sequence coverage and can be applied to complex samples, overcoming some of the shortcomings of bottom-up and top-down proteomics.^6^

**Figure 1:**
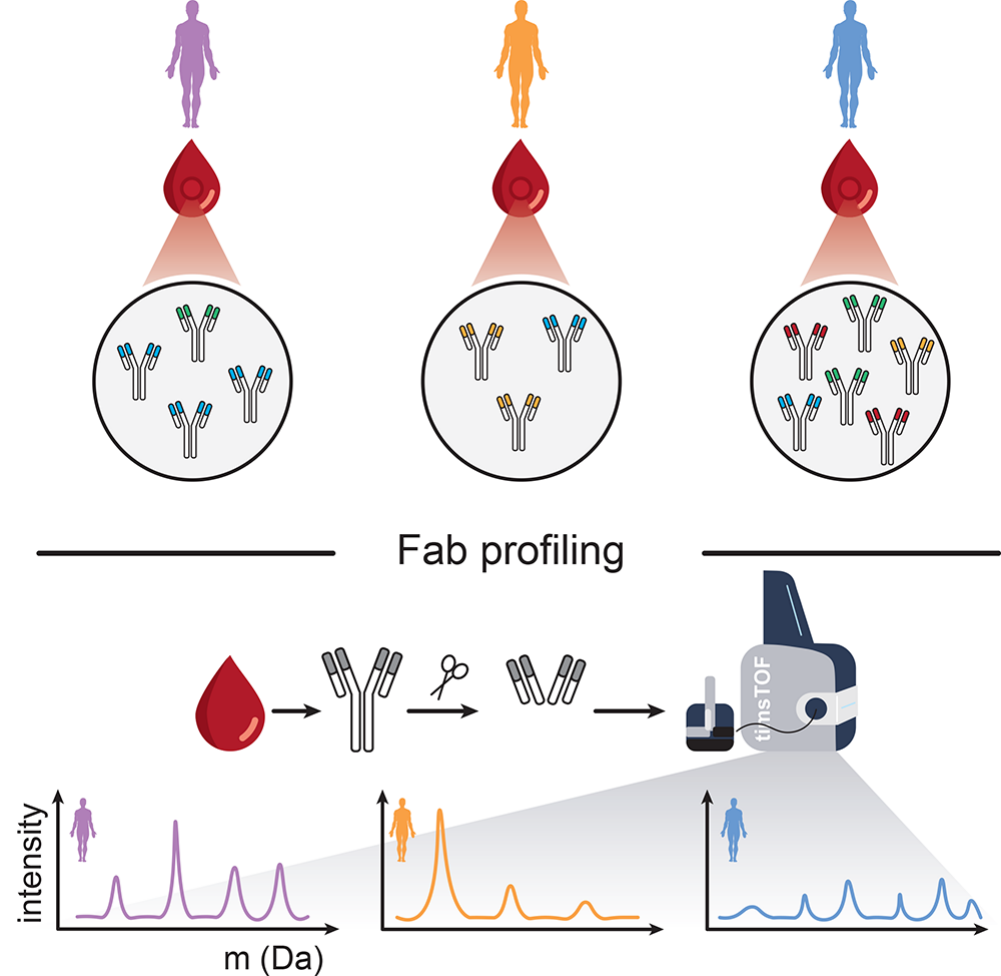
Antibody repertoire analysis through LC-MS based profiling of Fab fragments. For protein-centric Fab-profiling, IgGs are affinity enriched from serum obtained from individual donors. On the affinity-beads the Fab fragments are cleaved from the Fc parts (using a bacterial hinge-directed protease like IgdE) of all IgG1s. All Fabs originating from all IgG1s are subsequently separated and mass-analyzed by LC-MS on a timsTOF HT. The chromatography and timsTOF HT settings were optimized for the separation and analysis of intact Fab fragments (*M*_w_ ≈ 46–51 kDa). Each unique Fab fragment is characterized by a unique mass and retention time.

Recently, our group introduced and applied a mass-spectrometry (MS)-based approach that allows for quantitative, qualitative, and longitudinal studies of immunoglobulins of type G and A (subclass 1), IgG1 and IgA1, repertoires in plasma, serum, and breast milk samples.^7−9^ Briefly, this workflow involves affinity purification of IgG or IgA using isotype-specific affinity resins, followed by cleavage of Fab fragments and subsequent intact mass analysis by reversed phase LC-MS using a MAbPac (Thermo Scientific) column and Orbitrap-based instruments (Figure 1).

The resulting data would then be processed using BioPharmaFinder (Thermo Scientific) using the ReSpect algorithm. With this workflow, we demonstrated in earlier work that IgG1 clonal profiles are highly individual and unique to each donor; they remain stable over time but can change in response to immunological challenges.^8^ These unique clones exhibit differential responses to viral infections, such as the one caused by SARS-CoV-2, where in some cases the antigen-directed Ig population is dominated by only a few clones.^9,10^

All our previous Ig profiling studies were performed using Orbitrap-based mass analyzers.^7−11^ Notwithstanding the fact that Orbitrap-based instruments are among the most used instruments for peptide- and protein-based proteomics, alternative platforms have gained traction in recent years. Among these, the timsTOF series stands out especially for its use in high-throughput peptide-centric proteomics applications in, for instance, plasma proteomics^12^ and affinity-pull-down proteomics.^13^ timsTOF instruments are equipped with a time-of-flight (TOF) analyzer, in Q-TOF geometry, and additionally offer the possibility to perform high-resolution ion mobility analysis. While these platforms are already well established in the field of peptide-centric proteomics,^14^ fewer—but highly promising—studies have reported on the use of timsTOF instruments for analyzing intact proteins, or protein-centric proteomics.^15−17^ The potential of the timsTOF platform for intact protein analysis was demonstrated on ubiquitin already during its prototyping stage.^18^ Recent work showed that, with the help of a few hardware modifications, timsTOF instruments could be adapted even for native MS, analyzing intact protein complexes, notably GroEL (ca. 800 kDa).^15^ Due to some advantages of TOF over Orbitrap (and other) analyzers, i.e., high mass resolving power preserved at high *m*/*z* independently of scan time, timsTOF instruments have recently also been used for MS imaging of isotopically resolved intact proteins.^16^ A further interesting development has been the coupling of capillary electrophoresis to a timsTOF to analyze reduced monoclonal antibody fragments.^17^ The timsTOF instruments thus appear to be an interesting alternative to Orbitrap-based mass analyzers, also for our needs to profile personalized Fab repertoires.

Here, we report the progress we have made so far to analyze and profile Fabs to obtain information on the circulating Ig population in human serum samples using a Bruker timsTOF HT mass spectrometer. First, we adapted the chromatography and mass spectrometry settings to enhance the separation, ionization, and transmission of the intact Fab molecular ions throughout our analytical workflow, making use of the knowledge we obtained earlier using the Orbitrap-based workflows. Using a well-defined set of nanoLC settings and individual donor samples, we were able to compare the qualitative and quantitative Fab profiles obtained by using the timsTOF-based approach to those obtained in parallel using an Orbitrap Eclipse Tribrid platform. For an unbiased comparison, the timsTOF instrument was operated in MS only mode and without the trapped ion mobility analyzer. We show that similar data quality and profiling depth can be achieved with this setup, using the Bruker Compass DataAnalysis software, in combination with in-house-developed data analysis scripts, whereby the high sensitivity of the timsTOF allowed us to analyze IgG1 repertoires by injection of just 50 ng of protein, corresponding to 50 nL of serum. Showing that high-quality Ig profiling data can be achieved not only on Orbitrap-based platforms but also on other instruments opens new avenues for qualitative, quantitative, as well as longitudinal humoral immune response profiling, which could enhance the adaptation of this approach in clinical settings.

## Experimental Section

### Serum Sample Collection

Individual serum samples from healthy donors were provided by G. Vidarsson from Sanquin Research (Amsterdam, The Netherlands) and obtained in accordance with the ethics board of Sanquin and after informed consent from the donors. The samples were stored at −80 °C until used for processing. For method optimization, we used monoclonal antibodies (mAbs) that were provided to us as gifts by Roche (trastuzumab) and Genmab (7D8 and F59).

### Fab Preparation

#### Monoclonal Fab Mixture

Fab fragments were produced separately for three different recombinant monoclonal antibodies, trastuzumab, 7D8, and F59. The antibodies were digested by incubating 200 μg of each antibody with the IgdE hinge-cleaving enzyme overnight (>16 h), with a 10:1 (m/m) antibody to enzyme ratio at 37 °C in 150 mM phosphate buffer, pH 7 (50:50 monosodium and disodium phosphate), under agitation at 750 rpm. Fab fragments were then purified by (i) removing the Fc fragments by incubating with 50 μL of CaptureSelect FcXL slurry for 1 h at room temperature with agitation (750 rpm), then (ii) removing the His-tagged IgdE by incubating with 50 μL of Ni-bead slurry for 30 min at room temperature with agitation (750 rpm). The reference Fabs were then buffer exchanged into 150 mM ammonium acetate using 7K Zeba spin columns (2 cycles) and stored at −80 °C before use.

#### Serum Fabs

Fab fragments were produced following methods described previously in ref (8) with minor modifications. Briefly, 40 μL of CaptureSelect FcXL slurry was washed with 150 mM phosphate buffer (monosodium and disodium phosphate) and incubated with 100 μL serum and a mixture of two monoclonal antibodies (400 ng trastuzumab and 7D8 used as internal standards) for 1 h at room temperature with agitation (750 rpm). The samples were washed with 4 × 200 μL of phosphate buffer and digested with 150 U of the IgdE hinge-cleaving enzyme (FabALACTICA, Genovis). After overnight (>16 h) incubation at 37 °C at 750 rpm the Fabs were eluted. The samples were aliquoted and stored at −80 °C before the measurements.

### LC-MS

#### Liquid Chromatography

The Fab fragments were separated by nanoLC on an UltiMate 3000 (Thermo Fisher Scientific, Bremen, Germany), using a reversed-phase 150 μm × 150 mm, 4 μm, 1500 Å MAbPac column (Thermo Fisher Scientific, Bremen, Germany) heated to a temperature of 50 °C. Separation was achieved by using a 50 min gradient at a flow rate of 1 μL/min, with a mobile phase consisting of 0.1% HCOOH in Milli-Q water (A) and 0.1% HCOOH in CH_3_CN (B), with starting conditions of 90% A and 10% B, ramping up from 10 to 28% over 1 min, then from 28 to 38% over 50 min, then 38 to 95% over 1 min, finally 95% B was maintained for 2.5 min before restoring initial conditions.

#### Mass Spectrometry

Following nanoLC separation, the Fab fragments were analyzed on both a timsTOF HT (Bruker Daltonics, Bremen, Germany) and an Orbitrap Eclipse Tribrid (Thermo Fisher Scientific, Bremen, Germany) mass spectrometer, in MS1-only and positive ion mode.

On the timsTOF HT, analyses were performed using a CaptiveSpray ion source equipped with a 20 μm (inner diameter) emitter with a capillary voltage of 1450 V and a dry temperature of 180 °C, without applying in-source CID. Spectra were acquired with TIMS off and Focus mode on, in the 500–3000 *m*/*z* range at a spectral rate of 1 Hz.

On the Orbitrap Eclipse Tribrid, analyses were performed using a Nanospray Ion Source with a static spray voltage of 2000 V and an ion transfer tube temperature of 275 °C, with a source fragmentation energy of 15 eV. Spectra were acquired in Intact Protein mode, in the 500–3200 *m*/*z* range at an Orbitrap resolution of 7.5K, with a maximum injection time of 50 ms and AGC target of 800000 (200%).

### Clonal Profiling Data Analysis

#### Processing of timsTOF Data

LC-MS chromatograms were processed using Bruker Compass DataAnalysis version 6.0 combined with in-house-written scripts. First, raw data were opened in DataAnalysis in which a script (written in Visual Basic) was run to perform a sliding window averaging method, followed by MaxEnt mass deconvolution. Briefly, the LC-MS data were first partitioned (in the 10–50 min region of interest) into slices of 0.3 min (18 s), with 0.05 min (3 s) of overlap between two consecutive slices. For each slice, the corresponding mass spectra were averaged, baseline subtracted, smoothed, and subsequently deconvoluted with MaxEnt to give intact mass information. Baseline subtraction was performed with a flatness level of 0.8. Smoothing was done with one iteration using a Gaussian smoothing algorithm with a smoothing width of 0.2 *m*/*z*. MaxEnt was run within an *m*/*z* range of 500–3000 Th, set to a low mass of 45000 Da, a high mass of 52000 Da, data point spacing of 0.1 *m*/*z*, and instrument resolving power of 10000. The resulting deconvoluted spectra were then exported as profile xy and centroid mgf files in which exported mass lists were determined by the Sum Peak finder algorithm with resolving power set to 6500 *m*/Δ*m*, peak finder version timsControl, and absolute intensity threshold of 500. Deconvoluted slices were merged into a single mass list with merging mass error set to 50 ppm with the in-house-developed tool HlxlToolchain.

#### Processing of Orbitrap Data

Acquired RAW files were processed by BioPharmaFinder version 3.2 (Thermo Scientific) utilizing the deconvolution algorithm ReSpect with sliding windows between retention time 10–50 min. Sliding window parameters were set to match the parameters mentioned in the timsTOF processing section. Merge tolerance of subsequent slides was set to 30 ppm with a maximal retention time gap of 2 min. The ReSpect deconvolution parameters were set as follows: peak model, intact protein; deconvoluted *m*/*z* range, 900–2400 Th; output mass range, 45000–52000 Da; targeted mass, 48000 Da; charge state range, 12–60; peak filter parameter, 95%. Deconvoluted profile mass spectra and compound lists were exported and used for downstream processing.

#### Similarity Scoring between timsTOF and Orbitrap Data

Similarity scoring was performed using the R package OrgMassSpecR.^19^ Briefly, for each Fab profile, the top 100 most intense clones were selected to give representative spectra. The different Fab-spectra were then compared in pairs using cosine similarity scoring (often used to compare MS/MS spectra), with a tolerance of 1.5 Da and without performing any further baseline correction. The resulting scores were thereafter used to build a correlation matrix for the entire data set.

## Results

### Evaluation of the Sensitivity of a timsTOF for Fab Analysis Using a Mixture of Monoclonal Antibodies

To assess and optimize the capability of a timsTOF and the data analysis workflow presented here for intact Fab analysis, initially an equimolar mixture of three different Fabs was prepared with known well-defined masses, derived from intact recombinant monoclonal antibodies (F59, trastuzumab, 7D8). This mixture was used to find the optimal settings for the transmission and detection of Fab fragments on the timsTOF.

**Figure 2:**
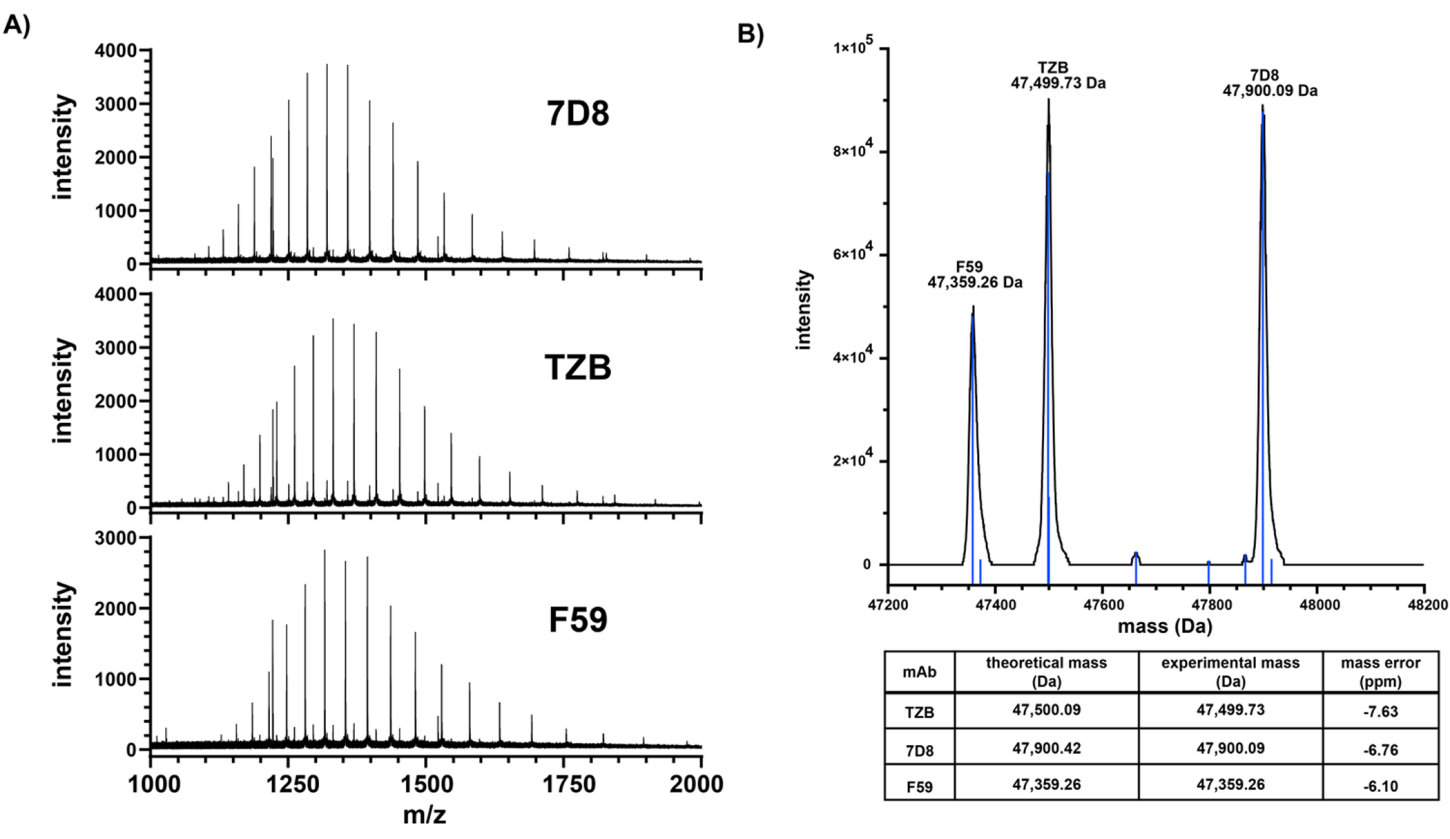
(A) Averaged ESI-mass spectra derived from nano-LC-MS runs of equimolar mixtures of Fab-fragments generated from the recombinant monoclonal antibodies (mAbs): 7D8, trastuzumab (TZB), and F59 (from top to bottom). The spectra shown represent typical charge state distribution (from 22+ to 42+) of intact, denatured Fabs measured on the timsTOF platform. (B) Deconvoluted zero-charge mass spectra obtained by using the sliding window processing pipeline presented in this work highlighting 3 major masses which correspond to theoretical masses of injected Fabs with an average mass error of ∼−6.8 ppm.

**Figure 3:**
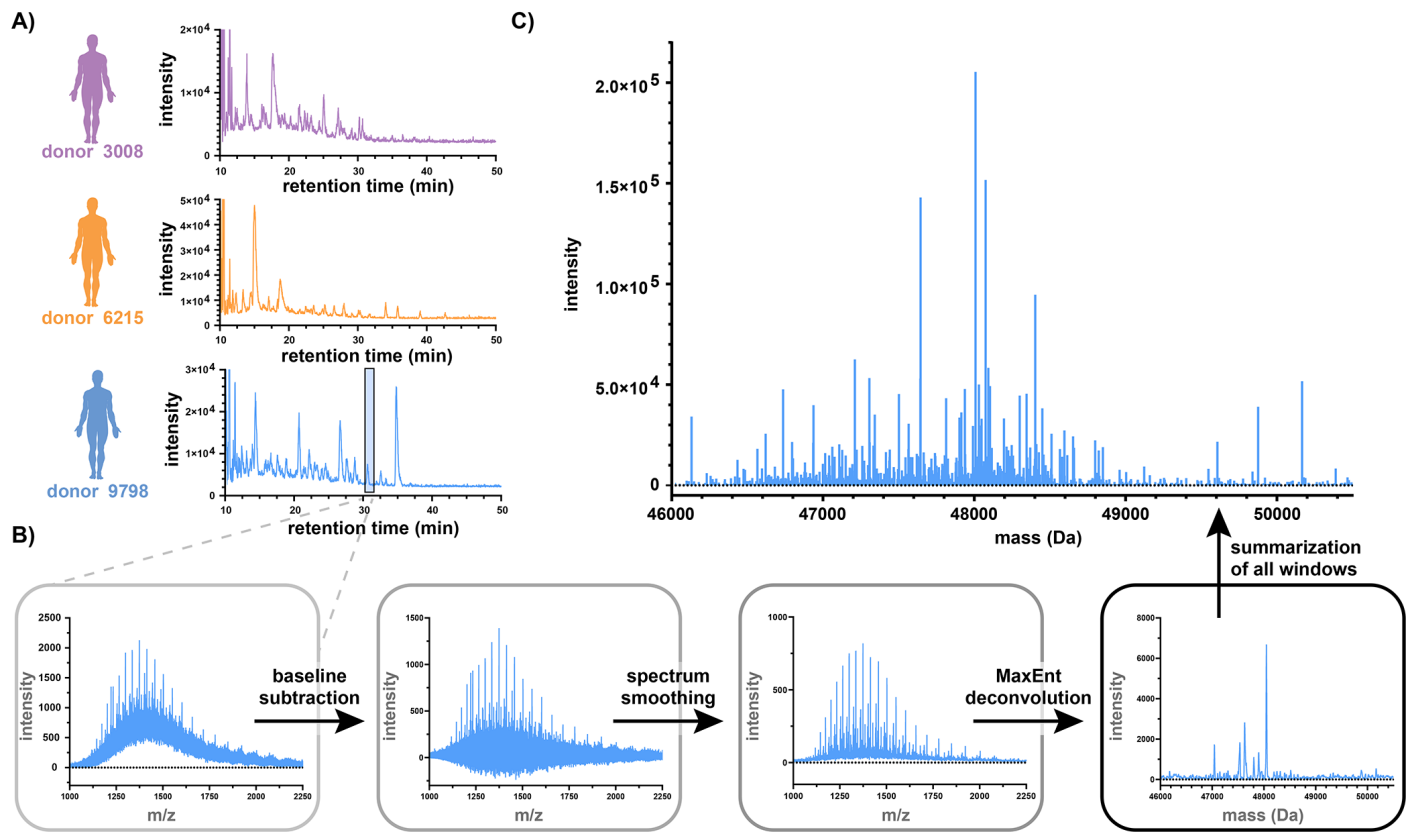
Conversion of LC-MS ion chromatograms to Fab-based antibody repertoires. (A) Base peak chromatograms of all Fab samples originating from the three different donors 3008, 6215, and 9798, respectively. (B) Flowchart presenting the preprocessing workflow for the serum samples, including integration of raw MS data, baseline subtraction, smoothing, and MaxEnt deconvolution (illustrated on a single slice for donor 9798). (C) Fab-clonal profile, resulting from the deconvolution of the full LC-MS ion chromatogram of donor 9798. The lines represent unique clones identified by peak picking each slice individually.

The Fab mixture, containing 20 nM of each Fab (corresponding to ∼240 pg per Fab for a 0.25 μL injection), was subsequently separated and analyzed on a nanoLC-timsTOF setup followed by data analysis in Bruker’s DataAnalysis software, complemented by in-house-written scripts (illustrated in Figure 3B). Using the optimized gradient, column, and mass analyzer settings, the three Fabs could be chromatographically well-separated and detected using the timsTOF, with charge state distributions ranging from *z* = 22 to *z* = 42 (Figure 2A).

To make sure that the data analysis workflow used would faithfully recapitulate the sample composition, this mixture of mAbs was analyzed with the same pipeline that was used for the subsequent analysis of donor serum samples. If the antibodies utilized for Fab generation were of high purity and our methodology was robust against the overinterpretation of noise, this sample should exclusively reveal the presence of three distinct Fabs. Indeed, the three Fabs contained in the mix were identified with an average absolute mass error of ∼6.8 ppm (Figure 2B). Additionally, a few more minor species were detected (all below 3% abundance), possibly being contaminations, carry-over from other samples, or post-translationally modified versions of the recombinant antibodies. From these LC-MS data on the Fab fragments derived from the mixture of three mAbs we conclude that after optimization of the settings sensitive separation and mass analysis of Fab fragments is achievable using the timsTOF platform, whereafter we moved next to the analysis of much more complex samples, directly extracted from the serum of single donors.

### Fab Analysis of Donor Serum on a nanoLC-timsTOF Platform Recapitulates the Complexity of Human Antibody Repertoires

In our initial studies on antibody repertoires, using Orbitrap-based platforms and microflow LC, we injected sample amounts corresponding to 2–4 μL of plasma or serum per injection and could then typically identify ∼250–550 distinct Fab clones.^20^ The current study demonstrates that moving toward nanoLC-MS scale has potential benefits such as lower sample consumption, better separation efficiency, improved ionization efficiency, and could therefore lead to deeper insight into antibody repertoires.^21^ For peptide-centric proteomics using nanoLC setups, the optimal sample amount injected into the system is in the range of tens of nanograms. Thus, for this pilot analysis, 0.25 μL injections of 10× diluted samples of intact Fabs generated from donor sera (corresponding to 56–110 ng, based on 280 nm absorbance) were tested on the nanoLC-timsTOF platform.

The collected data are presented in Figure 3A in the form of LC-MS base peak chromatograms for each donor. Each chromatogram is composed of a heterogeneous mixture of chromatographic peaks with a broad range of intensities, unique to each donor. To identify unique clones from this heterogeneous mixture, we make use of a sliding window charge state deconvolution approach (Figure 3B). In the raw TOF-MS data, the presence of an elevated baseline was observed in the 1000–2000 *m*/*z* range. We hypothesize that this is caused by the presence of a high number of lower-abundance unresolved Fab clones rather than actual noise. However, the available processing algorithms were not able to individualize all the low-abundance Fabs from this noise-like phenomenon; hence, we introduced a baseline subtraction in the following step. An additional step of smoothing afforded deconvoluted spectra with low levels of “noise” or signal overlap, allowing the identification of an average of 23 clones per sliding window. The number of detected clones per slice are depicted in Figure S1 (for donor 9798) and largely correlate to the total ion current of the nanoLC-MS chromatogram. Performing a peak picking step on every slice and summing them into a single graph afforded qualitative and quantitative plots representative of the Fab repertoires for each donor (e.g., Figure 3C for donor 9798). This allowed us to dissect several hundreds of unique clone masses for every donor: 820 ± 24 clones for donor 9798, 746 ± 28 for 3008, and 895 ± 20 for 6215 (see Table S1). The injected sample amount (0.25 μL ≡ 56–110 ng of Fabs) corresponds to merely 50 nL of input serum. Considering that 1–2 μL of blood is the suggested volume of a finger prick,^22^ these data indicate that antibody repertoire profiling could become amendable for immune response monitoring, without too much discomfort to patients. As depicted in Table S1 the number of identified clones could be even further enhanced to around ∼1100 and ∼1300 clones, with the injection of 1 (224–440 ng) or 5 μL (1.12–2.20 μg), respectively. These latter numbers are substantially above the highest number of detected clones per run we have been able to dissect using established Orbitrap-based platforms.

Although we detected most clones with the timsTOF when injecting 5 μL, we decided to continue by injecting 0.25 μL of a 10× diluted sample, as this provided still excellent data at the highest sensitivity (as illustrated by the base peak chromatograms for the different injection volumes in Figure S2). We next proceeded to acquire a complete data set for all the donor samples in triplicate and evaluated the quality and reproducibility of the corresponding Fab profiles on timsTOF and Orbitrap platforms.

### Benchmarking the Performance of the nanoLC-timsTOF Platform versus a nanoLC-Orbitrap-Based Setup

**Figure 4:**
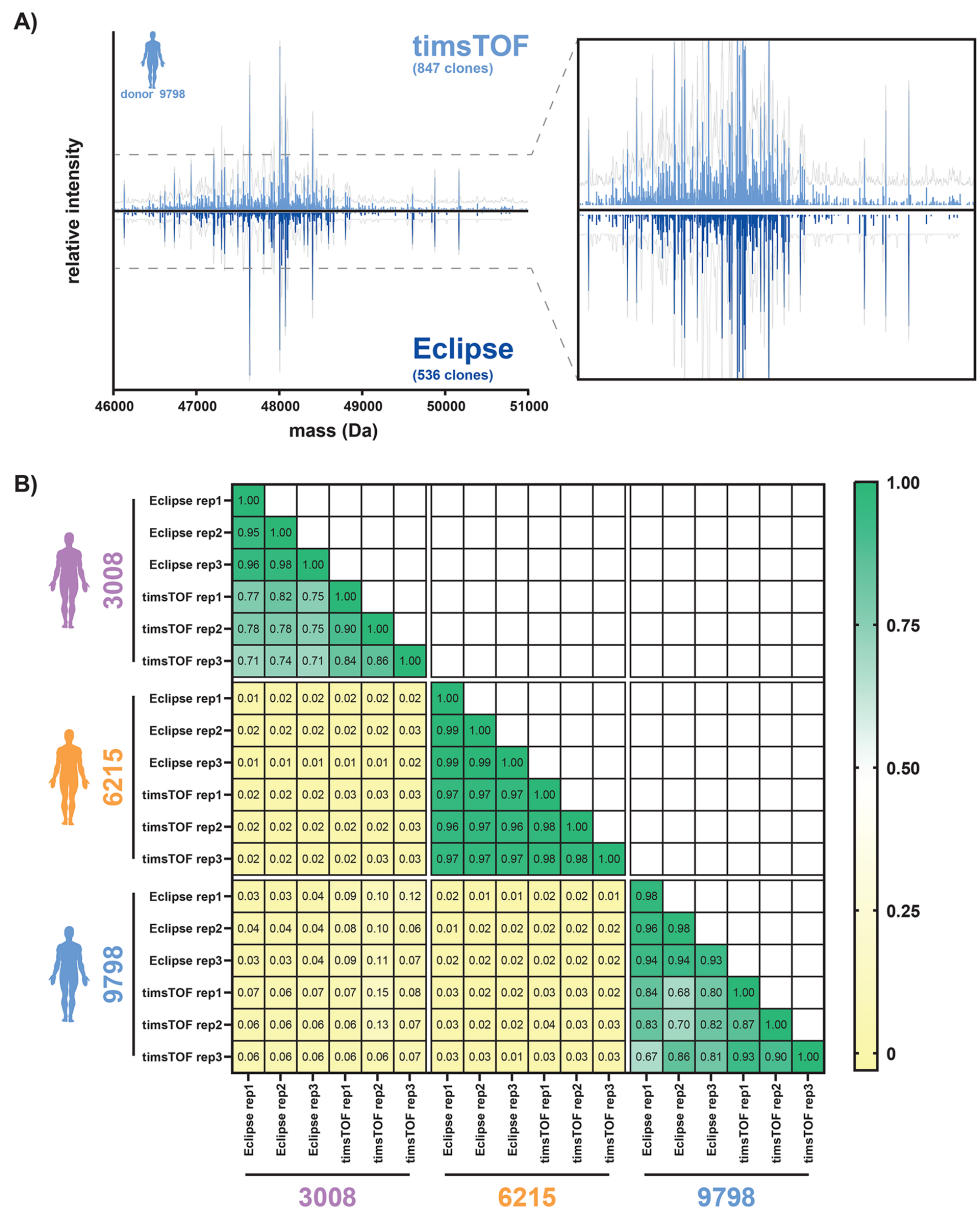
Comparison of Fab profiles obtained by using a timsTOF HT or an Orbitrap Eclipse Tribrid based platform. (A) Mirror plot of the Fab profiles obtained by timsTOF (top, light blue) and Eclipse (bottom, dark blue) for donor 9798. The number of identified clones is shown below the instrument name. The lines represent unique clones identified by peak picking each slice individually, and the gray profile represents the sum of all slices. Insert: zoom-in on the 0–35% intensity region. While the number of identified clones differs between platforms, the clonal profiles are very similar. (B) Cross-platform correlation heatmap for all 3 donors. Correlations were established using a cosine similarity score among the top 100 most intense clones of each Fab profile. Scores range from 0 (pale yellow) to 1 (green), with 0 indicating no similarity between the spectra and 1 indicating perfect similarity. Different donor samples show low correlation between themselves and across different platforms, whereas the same donor samples show good correlation between triplicate samples and the different platforms.

Having demonstrated that the timsTOF is a suitable platform to analyze complex mixtures of serum Fabs, we next aimed at comparing the data obtained with this new workflow to that obtained with Orbitrap instruments. The same samples were therefore analyzed on an Eclipse Tribrid instrument using the exact same UltiMate 3000 nanoLC setup as that used on the timsTOF, again injecting 0.25 μL of sample. Pleasingly, we observed very alike profiles in terms of clone masses and intensities (as depicted in Figure 4A for donor 9798 and in Figure S3 for donors 3008 and 6215). The deconvolution of the timsTOF data yielded a higher number of clones than Eclipse data, in the order of >1.5 times more clones (e.g., 847 unique clone masses for timsTOF HT and 536 for Eclipse for donor 9798), with the most notable difference being that we detected more low-intensity clones, as well as clones with slightly higher masses (Figure S4) on the timsTOF.

A straightforward way of evaluating the reproducibility of the Fab profiles across platforms is to compare the unique mass values and their corresponding intensities of the Fab fragments present in each sample, for instance, here using a cosine spectral similarity score (calculated from 0 to 1, 1 being a perfect match), which accounts for both clone mass and intensity. For the presented Fab profiling data, most of the overall signal intensity consisted of high-intensity clones, the top 100 clones representing over 50% for all timsTOF and Eclipse analyses, and even ∼75% of the total for Eclipse (Figure S5). The correlation score was therefore calculated for the 100 most intense clones in each sample with a tolerance of 1.5 Da for matching two clones together. Similarity scoring was performed for all possible pairs in our data set, presented as a correlation heatmap in Figure 4B. This evidenced not only a striking similarity across technical replicates on the same platform (profiles presented in Figure S6) but also a high correlation across the two platforms when analyzing samples of the same donor, with correlation scores ranging between 0.67 and even up to 0.97 between timsTOF and Orbitrap data. In contrast, when comparing the repertoire Fab profiles between different donors the correlation scores were, as previously shown, very low (in the range of 0.01–0.15), confirming the uniqueness of individuals’ repertoires.^8^ In summary, the great similitude in the data acquired on the timsTOF and the Orbitrap-Eclipse indicates that the very complex highly donor-specific Fab repertoires can be assessed independently of the MS platform used.

## Conclusion

Through the work described here, we demonstrate that (i) timsTOF mass spectrometers coupled to nanoLC can be used to accurately measure the mass of intact Fab fragments (*M*_w_ ≈ 46–51 kDa), from very low amounts of starting material down to <250 pg, (ii) this sensitivity can be exploited to generate Fab mass profiles from low amounts of serum samples by running a sliding window deconvolution on the nanoLC-MS data (as done in previous workflows using Thermo Scientific instruments and software), and (iii) the Fab profiles generated from such timsTOF data are alike to those that were generated by using Orbitrap instruments. Not only does this show that antibody repertoires can be explored independently of the MS platform, but this also demonstrates the capabilities of timsTOF instruments for protein-centric studies of complex biological samples.

Although it is always hard to compare the performance of different mass spectrometry platforms, even when using identical samples and LC setups, we are very pleased with the high number of individual clones we can detect per run on the timsTOF, which exceeds ∼1300 when injecting 5 μL. This high sensitivity of the timsTOF may be soon exploited to test a multidimensional chromatographic separation of the Fabs, for instance, by splitting a 5 μL sample into 20 differently separated 0.25 μL fractions, allowing likely a gain in depth of the monitored antibody profile.

We hypothesize that future incorporation of advanced top-down capabilities on the timsTOF may make this platform a very valuable alternative for protein-centric proteomics and top-down proteomics and thus also for personalized antibody repertoire profiling. Another future direction would also be to develop a standardized and user-friendly processing workflow to generate Fab profiles from timsTOF data, as this proof-of-concept study still uses a combination of different processing software and in-house scripts. Finally, it would also be valuable to incorporate the IMS capabilities of the timsTOF into the Fab profiling workflow, as this may help resolve additional species that are not separated with standalone LC-MS methods and thus study the human antibody repertoire in even more depth. Indeed, ion-mobility-based methods such as collision-induced unfolding have proved extremely useful to characterize intact antibodies in terms of disulfide bonding or glycosylation patterns,^23^ or even to study the higher-order structure of bispecific antibodies.^24^

## Supporting Information Available

Details on the number of Fabs detected for each injection, base peak chromatograms for different injection volumes, representative Fab profiles on timsTOF and Eclipse for donors 3008 and 6215, summed fractional intensities of all identified clones in each analysis, and Fab-clonal profiles for every replicate and donor (for 0.25 μL injections on timsTOF and Eclipse) (PDF)
