# Supplementary material for: Protein-Centric Analysis of Personalized Antibody Repertoires Using LC-MS-Based Fab-Profiling on a timsTOF
Source: J Am Soc Mass Spectrom. 2024 Apr 25;35(6):1292–300. doi: 10.1021/jasms.4c00076 (PMC11157643; doi:10.1021/jasms.4c00076)
Supplement: Supplementary file 1 — js4c00076_si_001.pdf [file js4c00076_si_001.pdf]

Supplementary information for:

Protein-centric analysis of personalized antibody repertoires using LC-MS-based Fab-profiling on a timsTOF

Jan Fiala[1,2‡], Dina Schuster[1,2‡], Simon Ollivier[1,2‡], Stuart Pengelley[3], Markus Lubeck[3], Florian Busch[4], Andris Jankevics[1,2†], Oliver Raether[3], Jean-Francois Greisch[4], Albert J. R. Heck[1,2*]

[1]Biomolecular Mass Spectrometry & Proteomics, Bijvoet Center for Biomolecular Research & Utrecht Institute for Pharmaceutical Sciences, Utrecht University, Padualaan 8, 3584 CH Utrecht, The Netherlands.

[2]Netherlands Proteomics Center, Padualaan 8, 3584 CH Utrecht, The Netherlands

[3]Bruker Daltonics GmbH & Co. KG, Fahrenheitstrasse 4, 28359 Bremen, Germany

[4]Bruker Switzerland AG, Fällanden, Zurich, Switzerland

‡These authors contributed equally. *Corresponding author: a.j.r.heck@uu.nl

†Present address: Department of Biochemistry, Institute of Systems, Molecular & Integrative Biology, University of Liverpool, Liverpool, UK

Table of Contents

Supplementary Table S1. Total number of Fab clones detected for each injection 2

Supplementary Figure S1. Evolution of the number of clones per slice with retention time 3

Supplementary Figure S2. Injection volume optimization 3

Supplementary Figure S3. Mirror plot of the Fab profiles 4

Supplementary Figure S4. Distribution of the clone masses for each instrument.................................5

Supplementary Figure S5. Assessing Reproducibility of the data 6

Supplementary Figure S6. Fab-clonal profiles of the donors recorded on either the timsTOF or the Orbitrap Eclipse mass analyzers...7

injection volume	donors 3008	6215	9798
0.25 µL	776	914	847
	722	874	812
	740	897	802
1 µL	986	1095	1130
	1031	1109	1126
	1047	1092	1148
5 µL	1235	1383	1393
	1306	1402	1395
	1137	1246	1253

timsTOF

donors 3008	6215	9798
510	531	536
489	512	492
405	409	420

Eclipse

Supplementary Table S1. *Total number of Fab clones detected following injection of different sample volumes (0.25, 1, 5 µL) on the timsTOF HT and 0.25 µL on the Orbitrap Eclipse platform, in triplicate for each of the three donors. Analyses on the timsTOF HT resulted in a higher number of detected clones compared to the analysis on the Orbitrap Eclipse, using an alike injection of 0.25 µL. Injection of more material (1 and 5 µL), evaluated only on the timsTOF HT, yielded to even higher number of detected clones. However, higher injections volumes seemed to negatively influence the performance of nanoLC separation (chromatographic peaks resolution, peak shape of highly abundant clones).*

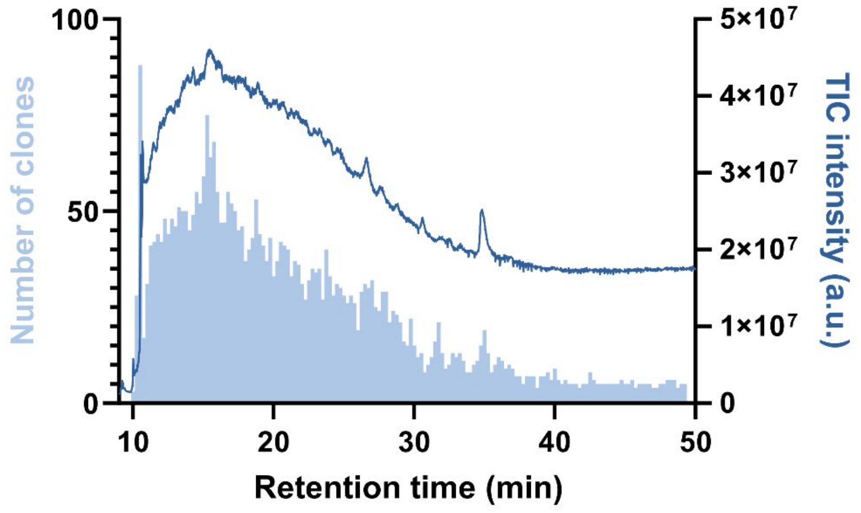

Supplementary Figure S1. Evolution of the number of clones per slice over the LC retention time. The number of clones per slice (bar graph, light blue) correlates with the intensity of the total ion current (profile, dark blue) across the nanoLC-MS chromatogram, indicating that the results of the MaxEnt deconvolution follow an alike trend as the raw data. The depicted data is from an analysis on a sample of donor 9798 (replicate 1).

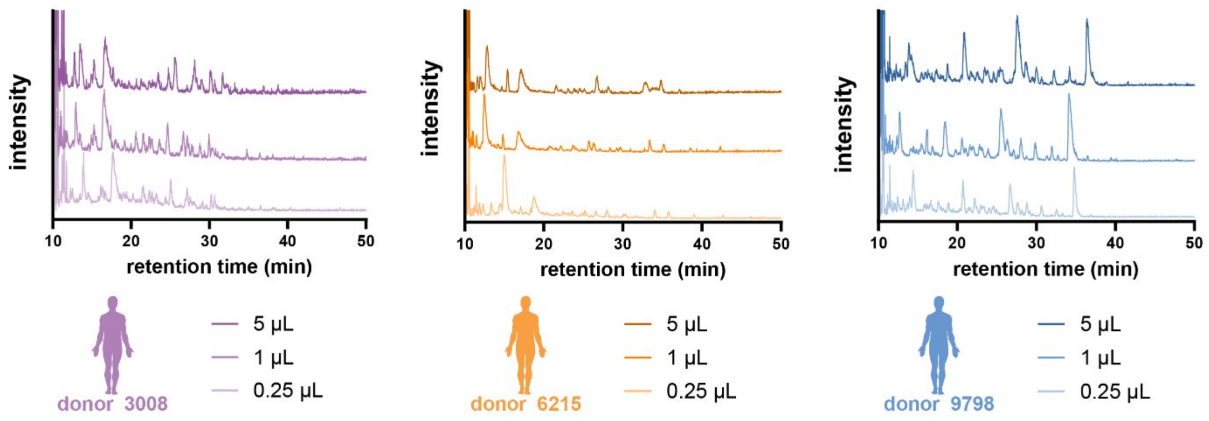

Supplementary Figure S2. Optimization of *injection volume. Illustrative base peak ion-chromatograms for the injected Fab samples originating from each of the three donors, acquired at different injection volumes (5, 1 and 0.25 µL of a 1:10 dilution of the Fab eluates, respectively).*

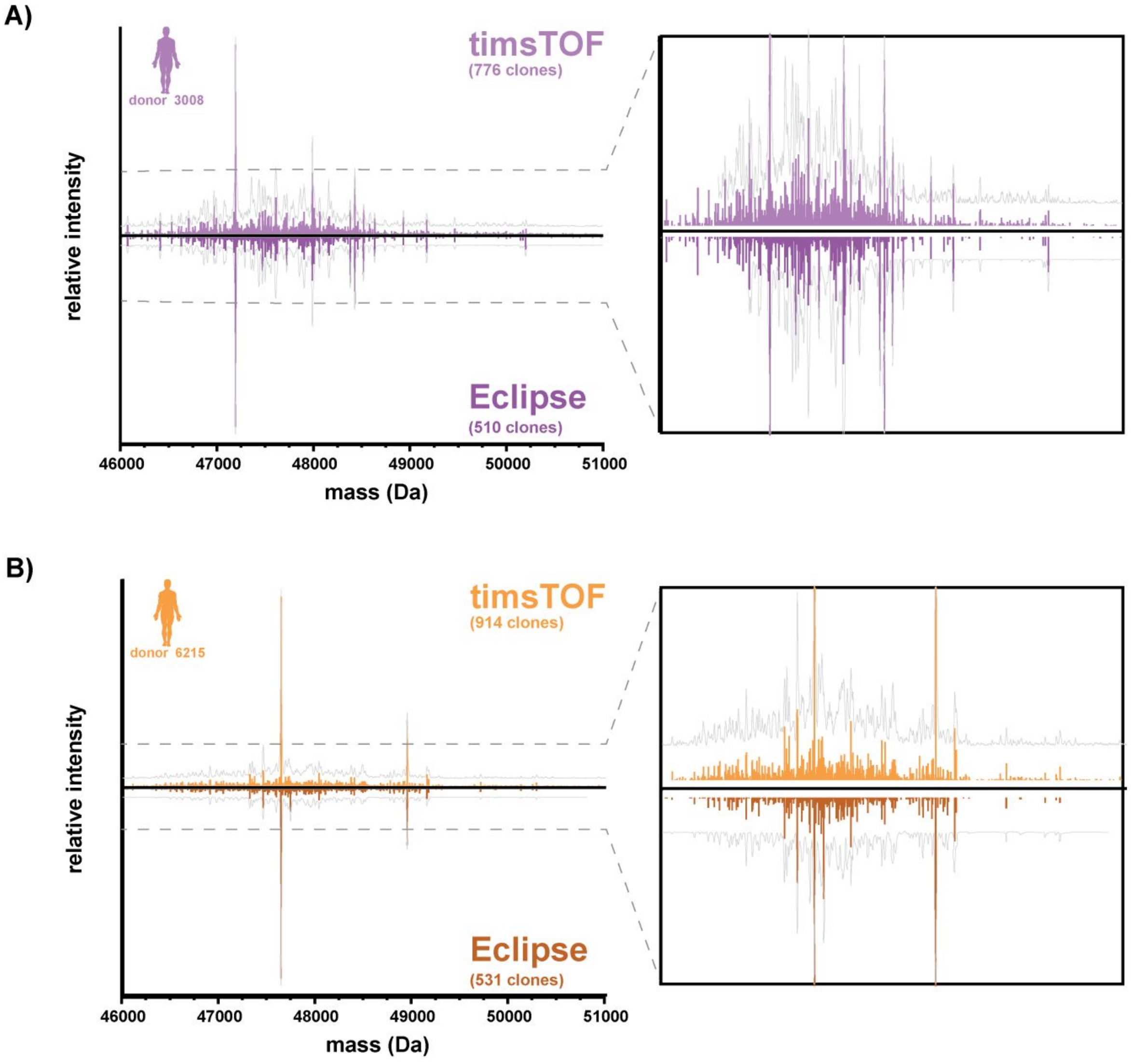

Supplementary Figure S3. *Mirror plot of the Fab profiles (46000-51000 Da) obtained from 0.25 µL injections of the 1:10 diluted Fab eluates recorded by using the timsTOF (top) and Orbitrap Eclipse (bottom) for donors 3008 (purple) (**A**) and 6215 (orange) (**B**), complementing the data shown for donor 9798 in the main Figure 4A. Each vertical line represents a unique clone identified by peak picking each slice individually, the gray profile represents the sum of all slices. The inserts are zoom-ins on lower-intensity regions. The Fab profiles obtained using a timsTOF and an Eclipse Tribrid were also very similar for the donors 3008 (Figure S2A) and 6215 (Figure S2B).*

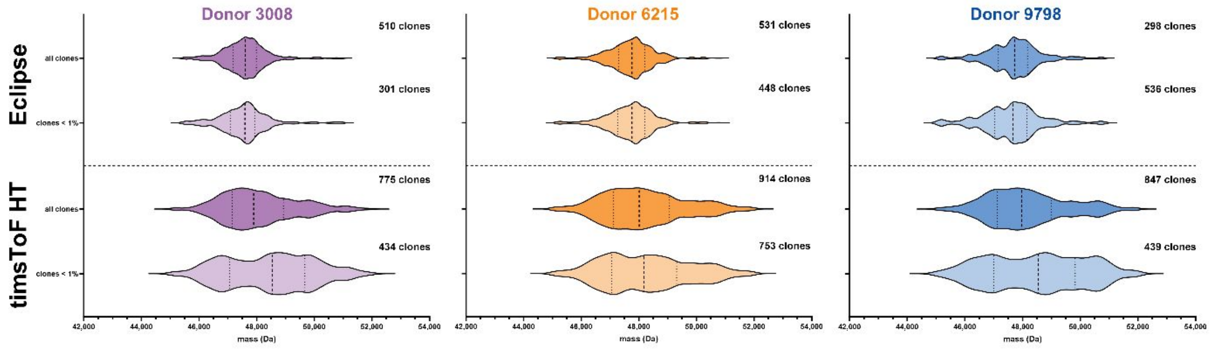

Supplementary Figure S4. *Binned mass distribution of the clones as detected with the two different mass analyzers. Mass distribution violin plots of all clones and clones with abundance lower then 1 % identified by Eclipse (Top) and timsTOF HT (bottom) for donors 3008 (purple), 6215 (orange) and 9798 (blue). Compared to the Eclipse data, the timsTOF data shows more consistently low abundant clone distribution through entire mass range.*

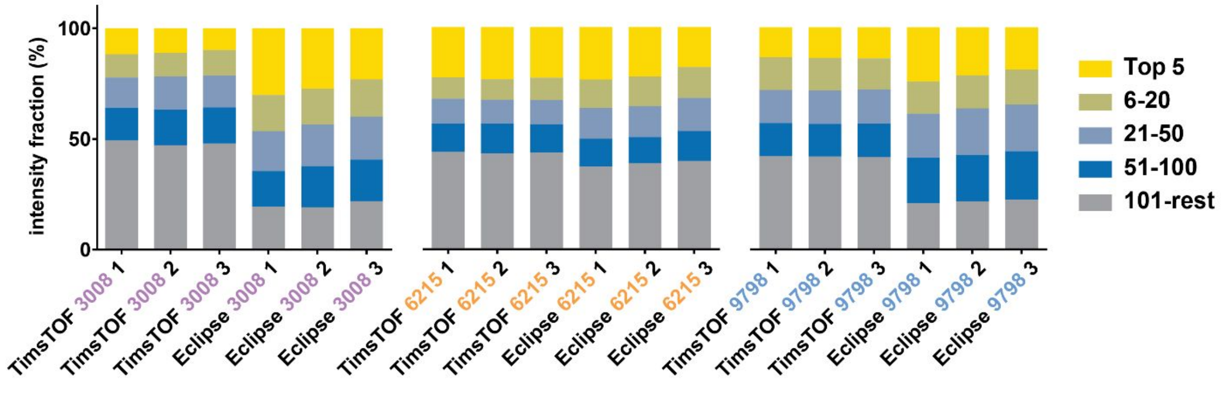

***Supplementary Figure S5**. Assessing Reproducibility of the data. Stacked bar plots of the summed fractional intensities of all identified clones. The top 5 clones are depicted in yellow, the top 6-20 in green, the top 21-50 in light blue, the top 51-100 in dark blue, the remainder of the clones is depicted in grey. The height of the individual colored bars represents the sum of the clonal intensity percentages ((clone intensity / sum of all intensities)*100), summed up for the respective fractions (i.e. top 5, 6-20, etc.). For every donor, the top 5 most abundant clones identified in the 0.25 µL injections of 1:10 diluted Fab elutions represented ~10-30% of the fractional abundance (calculated by dividing the individual intensities by the total summed intensity of clones identified in the respective sample), while the top 100 represented >50% of the total (**Figure S3**). The intensities of the different fractions were highly reproducible between different replicates on the same platform, with some sample-dependent differences between platforms.*

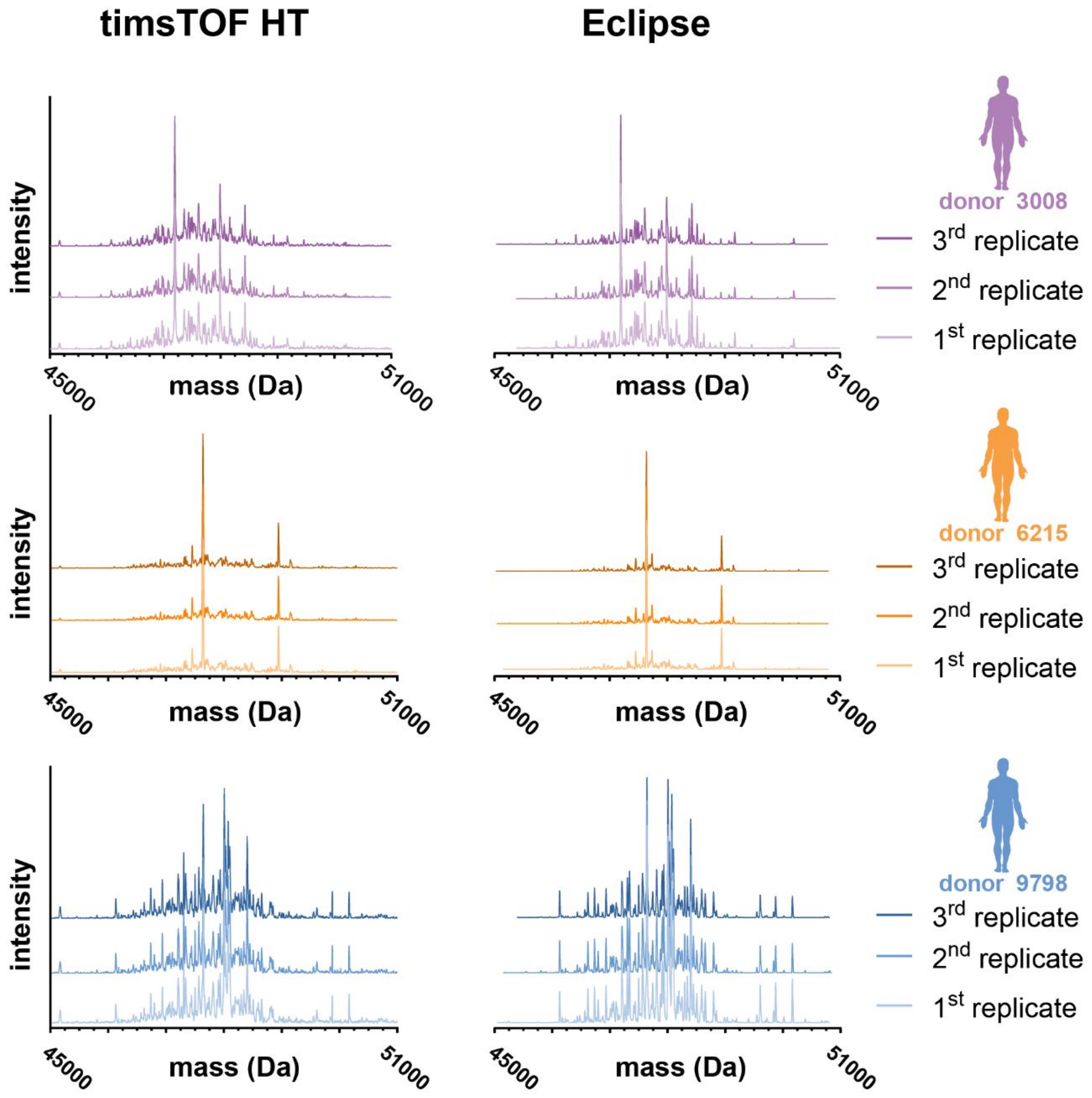

Supplementary Figure S6. *Fab-clonal profiles of the donors recorded on either the timsTOF or the Orbitrap Eclipse mass analyzers. The results from 3 replicates of 0.25 µL injections are depicted for each donor. TimsTOF HT results are shown on the left, Eclipse results on the right. As expected, the clonal profiles were highly donor-specific, the replicates were highly reproducible. Clonal profiles are also very similar across the two different platforms.*
